# Differential activity of 2-methylene-19-nor vitamin D analogs on growth factor gene expression in rhino mouse skin and comparison to all-*trans* retinoic acid

**DOI:** 10.1371/journal.pone.0188887

**Published:** 2017-11-28

**Authors:** Jamie M. Ahrens, James D. Jones, Nirca J. Nieves, Ann M. Mitzey, Hector F. DeLuca, Margaret Clagett-Dame

**Affiliations:** 1 Biochemistry Department, University of Wisconsin-Madison, Madison, Wisconsin, United States of America; 2 Pharmaceutical Sciences Division, School of Pharmacy, University of Wisconsin-Madison, Madison, Wisconsin, United States of America; University of Alabama at Birmingham, UNITED STATES

## Abstract

While all 2-methylene-19-nor analogs of 1α,25-dihydroxyvitamin D_3_ (1α,25(OH)_2_D_3_) tested produce an increase in epidermal thickness in the rhino mouse, only a subset reduce utricle size (comedolysis). All-*trans* retinoic acid (atRA) also causes epidermal thickening and a reduction in utricle size in the rhino mouse. We now report that 2-methylene-19-nor-(20S)-1α-hydroxybishomopregnacalciferol (2MbisP), a comedolytic analog, increases epidermal thickening more rapidly than does atRA, while both reduce utricle area at an equal rate. Whereas unlike atRA, 2MbisP does not alter the epidermal growth factor receptor ligand, heparin-binding epidermal growth factor-like growth factor, it does increase the expression of both amphiregulin and epigen mRNA, even after a single dose. *In situ* hybridization reveals an increase in these transcripts throughout the closing utricle as well as in the interfollicular epidermis. The mRNAs for other EGFR ligands including betacellulin and transforming growth factor-α, as well as the epidermal growth factor receptor are largely unaffected by 2MbisP. Another analog, 2-methylene-19-nor-(20S)-26,27-dimethylene-1α,25-dihydroxyvitamin D3 (CAGE-3), produces epidermal thickening but fails to reduce utricle size or increase AREG mRNA levels. CAGE-3 modestly increases epigen mRNA levels, but only after 5 days of dosing. Thus, 2-MbisP produces unique changes in epidermal growth factor receptor ligand mRNAs that may be responsible for both epidermal proliferation and a reduction in utricle size.

## Introduction

Skin is a vitamin D target organ [1–4]. The outermost skin layer, the epidermis, consists of a stratified layer of keratinocytes. Dividing keratinocyte stem cells in the basal layer continually give rise to cells with more restricted growth potential forming the upward columnar units of differentiating cells in the suprabasal layer [5, 6]. The hair follicles are appendages of the of epidermis that undergo cycles of regeneration from stem cells in the outer root sheath (bulge). The hormonal form of vitamin D_3_, 1α,25-dihydroxyvitamin D_3_ (1,25(OH)_2_D_3_), acts by binding to the vitamin D receptor (VDR) that is located in the nuclei of cells in the basal and suprabasal layers of the epidermis as well as in the hair follicle outer root sheath, and is largely absent from the dermis [1, 2, 7, 8]. 1,25(OH)_2_D_3_ and the VDR influence the proliferation and differentiation of keratinocytes [9–11]. The loss of VDR produces an abnormality in initiation of the hair cycle [12, 13]. 1,25(OH)_2_D_3_ and its analogs have been shown to differentially affect skin dependent upon the pathophysiological state or model system studied [14]. In cultured keratinocytes, 1,25(OH)_2_D_3_ and analogs can enhance or inhibit proliferation depending upon the selected culture conditions and hormone concentration [15–18]. In psoriasis, 1,25(OH)_2_D_3_ and analogs inhibit excessive proliferation of keratinocytes and promote differentiation [19, 20]. However, topical administration of 1,25(OH)_2_D_3_ and its analogs to the skin of normal humans [21] as well as some mice [22–26] can cause thickening of the epidermis.

Our group previously evaluated topical application of a series of 2-methylene-19-nor analogs of 1,25(OH)_2_D_3_ on skin of the rhino mouse. All 2-methylene-19-nor analogs tested induce a marked thickening of the epidermis and an increase in BrdU-labeling of basal cells [26]. Although the rhino mouse is hairless, it has utriculi which derive from the upper part of the original follicular unit, and are histologically similar to comedones found in human acne [27, 28]. Interestingly, a subset of the 2-methylene-19-nor analogs, specifically those with a shortened side chain and lacking a 25-hydroxyl group, are also effective at reducing the size of utricles (comedolysis). One such analog, 2-methylene-19-nor-(20S)-1α-hydroxybishomopregnacalciferol (2MbisP), produces both a reduction in utricle area and an increase in epidermal thickness [26]. In contrast, 2-methylene-19-nor-(20S)-26,27-dimethylene-1α,25-dihydroxyvitamin D3 (CAGE-3) containing a full side chain and a 25-hydroxyl group, increases epidermal thickness, but has no effect on utricle area. The underlying mechanisms responsible for the ability of 2MbisP and CAGE-3 to induce epidermal proliferation while at the same time producing differential effects on utricle size are unknown.

Epidermal proliferation is under the control of the epidermal growth factor receptor (EGFR) and its ligands [29, 30]. EGFR (ErbB1) is a member of the ErbB or Her family, a subclass of the receptor tyrosine kinase superfamily [31]. EGFR, ErbB2 and ErbB3 are expressed in human skin, with the EGFR being the predominant receptor in regulating ligand-dependent proliferation [32]. Ligands for the EGFR include amphiregulin (AREG), betacellulin (BTC), epidermal growth factor (EGF), epigen (EPGN), epiregulin (EREG), heparin-binding EGF-like growth factor (HB-EGF), and transforming growth factor-α (TGF-α). EGF binding to EGFR induces formation of receptor homo- and heterodimers and initiates multiple cellular signal cascades such as the Ras-Raf-MAPK pathway, which affect cellular growth. Keratinocytes produce EGFR ligands that regulate the proliferation of stem cells in the basal layer [33–36].

Retinoids are used clinically to treat a variety of human skin disorders including acne and psoriasis [37, 38]. In a mouse model of acne, retinoids induce a reduction in utricle size and an increase in epidermal thickness [28, 39–47]. Hyperproliferation of basal keratinocytes leading to epidermal thickening has also been observed in human skin *in vivo* following topical application of retinoids [48]. It has been suggested that the hyperproliferative effect associated with retinoid therapy is necessary to achieve the desired reduction in utricle size (comedolysis) [39].

Retinoids cause thickening of the skin by increasing proliferation of basal keratinocytes through activation of the EGFR signaling pathway. Topical application of all-*trans* retinoic acid (atRA) to normal mouse skin mediates gene transactivation resulting in release of the growth factor HB-EGF from suprabasal keratinocytes, whereas other EGFR ligand mRNAs including amphiregulin and TGF-α are not significantly altered [49]. The resulting increase in HB-EGF acts in a paracrine manner to bind and activate EGFR of basal keratinocytes and induce proliferation [36, 50]. Likewise, in normal human skin, topical application of atRA induces expression of HB-EGF mRNA [51, 52].

Herein, we evaluate EGFR ligand and EGFR (ErbB1) mRNA expression after topical exposure of mouse skin to the 2-methylene-19-nor analogs, 2MbisP and CAGE-3. Further, we compare the effects of the 2-methylene-19-nor analogs with those of atRA on mRNA expression and on skin morphology.

## Materials and methods

### Animals

All procedures involving animals were reviewed and approved by the University Committee on Use and Care of Animals at the University of Wisconsin—Madison, a facility accredited by the Association for the Assessment and Accreditation of Laboratory Animal Care International (AAALAC). Rhino mice (RHJ/LeJ), heterozygote female (Hr^rh-J^/^+^) and homozygote male (Hr^rh-J^/Hr^rh-J^) pairs, were obtained from Jackson Laboratory (Bar Harbor, ME, USA). This mouse carries a spontaneous recessive mutation of the hairless (Hr) allele with homozygous animals becoming hairless at about 3 weeks of age. Homozygous rhino mice (Hr^rh-J^/Hr^rh-J^) were generated from an in-house breeding program. They were housed in shoebox cages and maintained on a 12 h light/12 h dark cycle in the Department of Biochemistry vivarium. Mice were provided with autoclaved laboratory chow and water *ad libitum*. Homozygous rhino mice were assigned to study groups at 6 to 8 weeks of age. At study termination, animals were euthanized with carbon dioxide followed by destruction of vital organs.

### Chemicals

**Fig 1:**
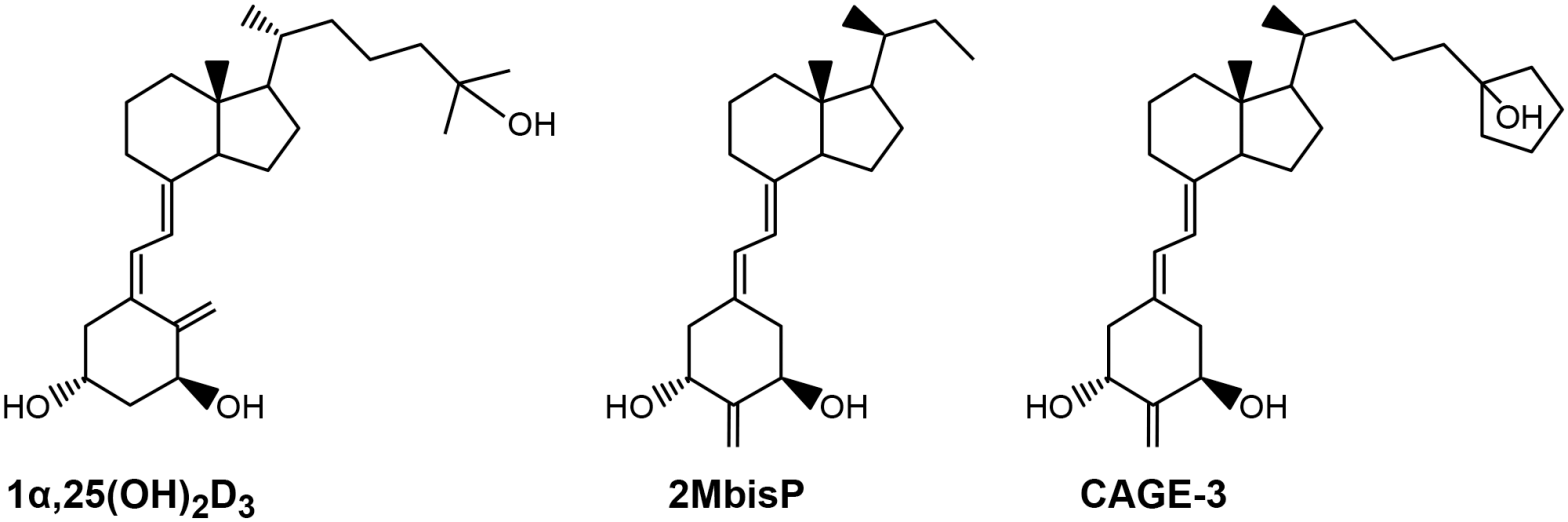
Structure of 1α,25(OH)_2_D_3_ compared to the 2-methylene-19-nor analogs, 2MbisP and CAGE-3.

The 2-methylene-19-nor analog, 2MbisP, was synthesized by SAFC (Madison, WI) [53] and CAGE-3 [54] was synthesized in the laboratory of Dr. Hector DeLuca (University of Wisconsin-Madison, WI). The structures are shown in Fig 1. atRA was purchased from Spectrum Chemical and Laboratory Products. Compounds were dissolved in 100% ethanol and their concentration determined by UV spectrophotometry using a molar extinction coefficient of 45,200 at 350 nm for atRA and 42,000 at 252 nm for the 2-methylene-19-nor analogs. 2MbisP and CAGE-3 were analyzed for purity by high pressure liquid chromatography using an analytical normal phase Zorbax SIL column using hexane and IPA at a ratio of 96%:4% (0–31 min) and a gradient to 90%:10% over 10 min at a UV absorbance maxima of 252 nm. atRA was analyzed by high pressure liquid chromatography as previously described [55]. The purity of 2MbisP, CAGE-3, and atRA was greater than or equal to 98%, 96% and 100%, respectively. Dosing solutions were prepared in an ethanol:propylene glycol vehicle (70:30, v/v) and were stored at 4°C.

### Treatment

The drug dose or vehicle alone was applied on the dorsal trunk over an area of approximately 2.5 cm by 5 cm in a volume of 3.3 μL per g body weight (equivalent to 100 μL for a 30g mouse). For studies lasting longer than 7 days, the dose of drug administered was adjusted weekly based on body weight. Drug was administered topically at 24h intervals, mice were euthanized 4h after the final dose, and the dorsal skin was removed. Skin to be used for RNA analysis was flash frozen in liquid nitrogen and stored at -80°C until analysis.

### Skin histology

Skin biopsies for histology (approximately 1 cm in length) were laid flat on wooden sticks and fixed overnight in 4% paraformaldehyde in PBS, dehydrated with increasing percentages of ethanol, and embedded in paraffin. Five-10 μm sections taken at 150 μm intervals were evaluated from each biopsy and were stained with Gill’s Hematoxylin & Eosin (H&E). Sections were imaged and analyzed using MetaMorph software (Molecular Devices, Downingtown, PA) as previously described [26]. The area of every utricle (comedone) in each of 5 slices was measured, and the average utricle area was determined for each mouse. Similarly, epidermal thickness was determined by measuring the thickness of the epidermis in the interfollicular regions. From these data, group means were determined and expressed as percent of vehicle (mean ± standard error of the mean). The average utricle area in vehicle-treated mice in these studies was 9425 ± 440 μm^2^ and the epidermal thickness was 23.4 ± 0.37 μm. A total of 6 animals (3M and 3F) were analyzed per treatment group.

### RNA isolation and quantitative analysis

Frozen skin samples were ground in dry ice using a coffee grinder. Approximately 200 mg of sample was homogenized in 2 mL TriPure isolation reagent (Roche Diagnostics) and total RNA was isolated according to the manufacturer’s protocol. RNA was quantified using a NanoDrop 1000 spectrophotometer (Thermo Scientific). Total RNA (1 μg) was reverse transcribed using the High Capacity cDNA Reverse Transcription Kit (Applied Biosystems) according to the manufacturer’s protocol. Quantitative RT-PCR (qPCR) analysis was performed using the LightCycler instrument (version 1.2, Roche Diagnostics) and the LightCycler FastStart DNA Master SYBR Green I kit (Roche Diagnostics). The primer sets used to generate qPCR control plasmids and for qPCR analysis are described in Table 1. The amplicons of all qPCR control plasmids were verified by sequence analysis. qPCR values were normalized to β-actin expression levels, and values are expressed as fold-change from the vehicle-treated samples. mRNA from 4 animals (2M and 2F) was analyzed per treatment group. To detect genomic contamination, murine β-actin primers that span an intron were used in conventional PCR (primers: 5’-GGTGGGAATGGGTCAGAAGG-3’ AND 5’-GTACATGGCTGGGGTGTTGA-3’; NCBI accession # NM_007393.5; 241 to 508 bp (primers generate a 268bp product from all cDNA samples; in samples with genomic DNA contamination, an additional 722bp product would be observed). By this method, all cDNA samples used in this study were assayed and determined to be free of genomic contamination.

**Table 1:** qPCR primers.

Gene	NCBI Accession #	Product (bp)	Sense Primer (5’—3’)	Antisense Primer (5’—3’)
**Amphiregulin**	NM_009704	194	CCAATGAGAACTCCGCTGCT	GGCATTTCGCTTATGGTGGA
**β-Actin**	NM_007393	390	TGTTTGAGACCTTCAACACCC	CGTTGCCAATAGTGATGACCT
**Betacellulin**	NM_007568	285	ACACAACCAGAACACCAGAAA	CCATGACCACTATCAAGCAGAC
**Epidermal Growth Factor**	NM_010113	204	GTGGGAAGTCTGTTGTTGGA	GTAGATTGGAGCTGGCTATCAG
**Epidermal Growth Factor Receptor**	NM_207655	170	CACTGCTGGTGTTGCTGACCGC	TTCCAAGTTCCCAAGGACCA
**Epigen**	NM_053087	307	GCACTGAGCGAAGAAGCAGA	TTAGCAATCCGACGCCAATC
**Epiregulin**	NM_007950	163	TTTTTGTCCCACTCCGTCAG	CTCCCCCTTTCCCAGAACAT
**Heparin-Binding Epidermal Growth Factor Receptor**	NM_010415	203	AGCAACCAGCAACCCTGAC	TCCCTAACCCCTTTCCTTTCTTC
**Transforming Growth Factor-α**	NM_031199	170	AAGTGCCCAGATTCCCACA	GCAGTGATGGCTTGCTTCTTC

### In situ hybridization and immunohistochemistry

Skin was fixed for 48h in 4% PFA in PBS, embedded in paraffin, sectioned at 4–5 μm and stored on slides in desiccant until use. For *in situ* hybridization studies, slides were processed manually using the RNAscope^®^ 2.5 Reagent Kit-Brown and HybEZ^®^ Hybridization System from Advanced Cell Diagnostics (ACD) [56]. Briefly, paraffin was removed from sections using a graded series of ethanol and xylene, followed by treatment of sections with hydrogen peroxide (10 min, room temp), antigen retrieval solution (15 min, 99°C with slides in a beaker on a hot plate), and protease solution (15 min, 40°C). A positive control probe to peptidylprolyl isomerase B (Ppib) was included on each slide. RNAscope probes were from ACD (Mm-Areg; Mm-Epgn; Mm-Hbegf; Mm-Ppib). Slides were counterstained with methyl green (0.04% in 0.01M sodium acetate, pH 4.0) and coverslipped with Cytoseal 60 (Thermo Scientific).

For VDR immunohistochemistry all steps were performed at room temperature unless otherwise stated using an antibody (D-6; Santa Cruz Biotech, 13133) previously characterized by Wang and colleagues [57]. Sections were deparaffinized, re-fixed in 10% buffered formalin phosphate, treated with hydrogen peroxidase (1% in TBS; 50 mM Trizma base, 150 mM NaCl; pH 8.4) and permeabilized in 1% Triton X-100 in TBS, followed by antigen retrieval for 20 min at 99°C in citrate buffer (pH 6.0; Vector Laboratories) and cooling at room temperature for 1.5 h. Sections were blocked in 5% non-fat dry milk (NFDM) in TBS with 0.05% Tween 20 for 1 h, followed by overnight incubation at 4°C with VDR antibody diluted 100-fold in TBS with 1% NFDM and 0.05% Tween-20. Sections were washed, and VDR antibody binding was detected using biotinylated anti-mouse IgG followed by streptavidin-peroxidase (Vectastain ABC HRP, Peroxidase Mouse IgG, Vector Labs) and incubation with 3,3’-diaminobenzidine tetrahydrochloride, cobalt chloride and hydrogen peroxide. Tissues were dehydrated and coverslipped with Cytoseal 60.

### Statistical analysis

Statistical analysis was performed in GraphPad Prism 6.0 (GraphPad Software, Inc., La Jolla, CA). For 2MbisP, CAGE-3, and atRA dose-response studies, a one-way analysis of variance (ANOVA) was used with Tukey’s *post hoc* multiple comparisons test to analyze data from epidermal thickness measurements, utricle area, and mRNA expression. For multigroup comparisons, a two-way analysis of variance (ANOVA) was used with Tukey’s *post hoc* multiple comparisons test to analyze data. Descriptive statistical results are presented as mean ± standard error of the mean. Statistical significance was defined as *P≤0*.*05*.

## Results

### Changes in utricle area and epidermal thickness are maximal by 7 days of topical 2MbisP administration

**Fig 2:**
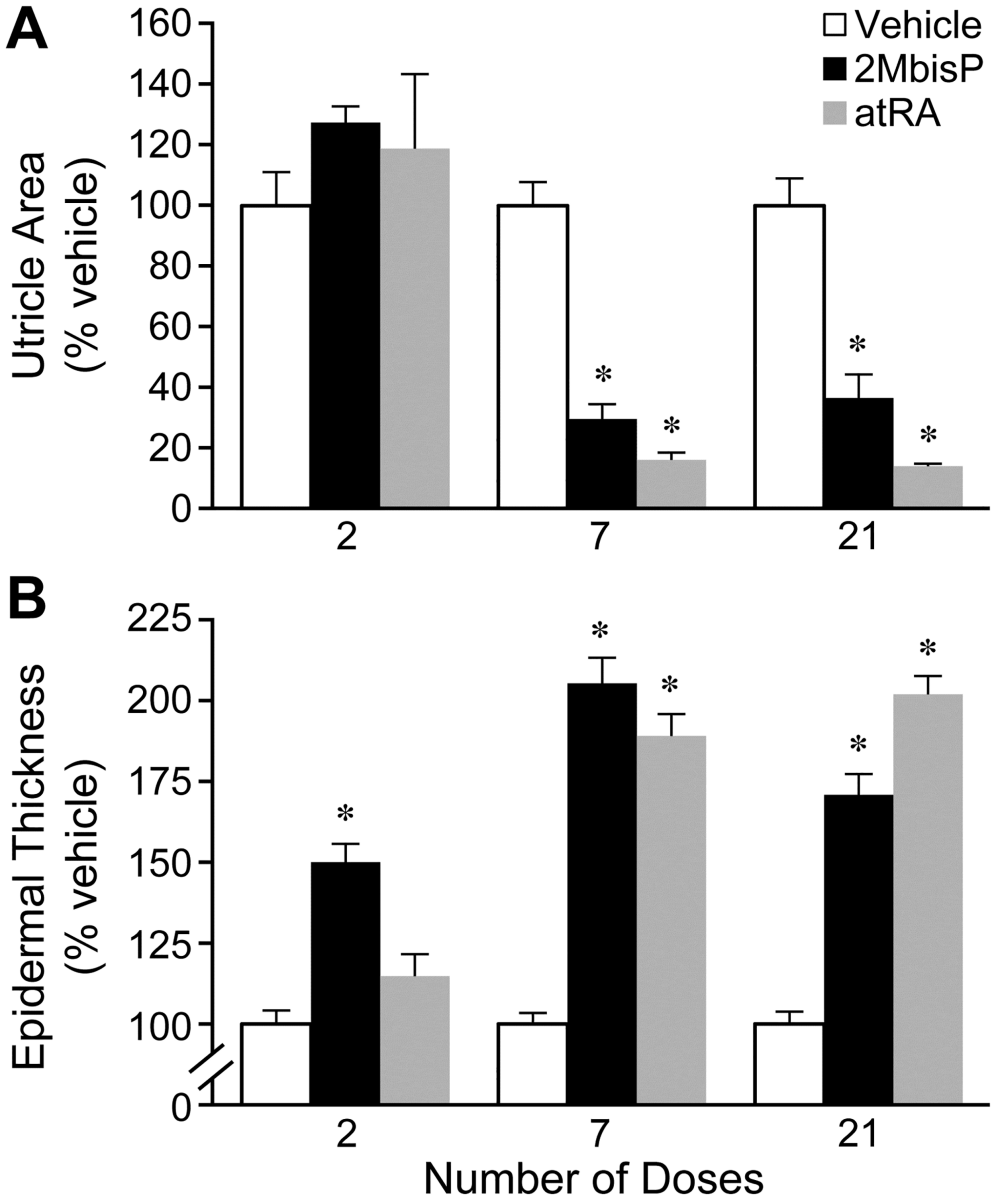
Effect of 2MbisP and atRA on utricle size and epidermal thickness after 2, 7 and 21 topical doses. (A) Utricle area and (B) epidermal thickness was measured in H&E stained tissue sections taken from mice receiving vehicle, 2MbisP (690 nmol/kg), or atRA (224 nmol/kg). The data are expressed as percent of vehicle. Significant differences from the vehicle group at each dosing time point are indicated by an asterisk, *P≤0.05 (S1 Table).

Previous work showed that the vitamin D analog, 2MbisP reduces utricle area (comedolysis) and increases interfollicular epidermal thickness after 21 days of daily topical application [26]. In an earlier study of atRA in the rhino mouse, hyperproliferation and comedolysis were reported after three weeks of topical treatment [42]. Closing of comedones after 6 days of treatment with atRA was observed by Zheng and colleagues [46]. In order to examine the time required for 2MbisP to act, animals were treated topically with 2MbisP (690 nmol/kg) and compared to atRA (224 nmol/kg) after 2, 7 or 21 daily doses, and skin morphology was examined. 2MbisP produced a significant reduction in utricle size after 7 doses that was equivalent to that seen after 21 doses (Fig 2). atRA produced a similar reduction in utricle area that was also maximal after 7 doses. 2MbisP, unlike atRA, produced a significant increase in epidermal thickness after only 2 doses. The effect was maximal and similar in magnitude for both compounds after 7 doses.

**Fig 3:**
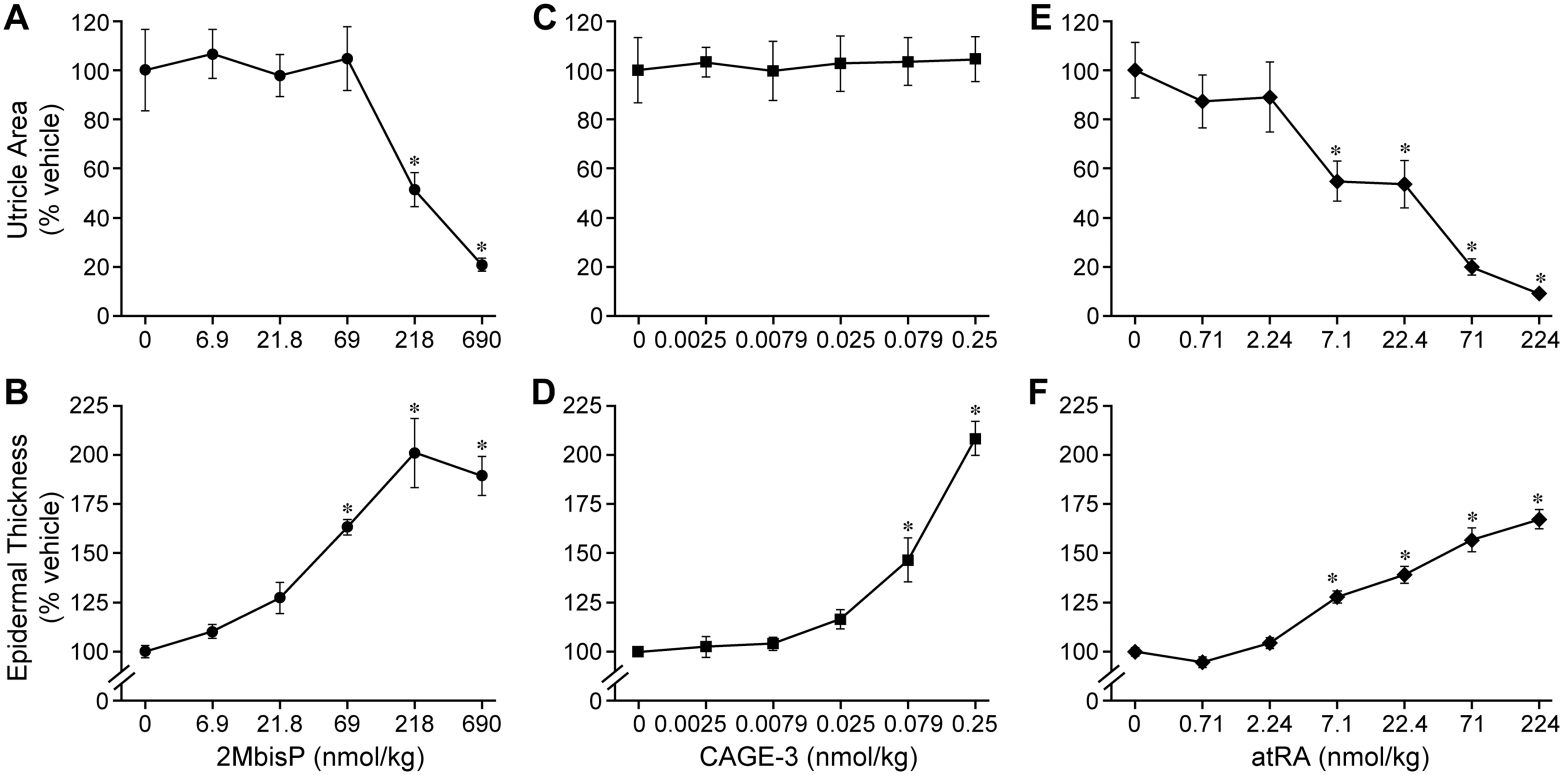
Dose response for 2MbisP, CAGE-3 and atRA on utricle area and epidermal thickness. Utricle area and epidermal thickness were measured in H&E stained tissue sections after the application of 7 doses of vehicle or varying doses of 2MbisP (A, B), CAGE-3 (C, D), or atRA (E, F). Significant differences from the vehicle group at each respective dose are indicated by an asterisk, *P≤0.05 (S2 Table).

Dose-response studies of 2MbisP, CAGE-3, and atRA on changes in utricle area and interfollicular epidermal thickness were conducted. After 7 doses, 2MbisP at 218 nmol/kg produced a 50% reduction in utricle area that was further reduced to 20% of vehicle at 690 nmol/kg (Fig 3). A small but significant increase in epidermal thickening was observed at 69 nmol/kg, with a maximal response occurring at 218 nmol/kg. CAGE-3 produced a significant increase in epidermal thickness at both 0.079 and 0.25 nmol/kg body weight, however, utricle area was unchanged at all doses tested. It was not possible to test higher doses of CAGE-3 due to dose-limiting hypercalcemia. At 7.1 nmol atRA/kg, utricle area was reduced by ~50% and was further reduced to ~10% that of the vehicle control at 224 nmol/kg. A small but significant increase in epidermal thickness was observed at 7.1 nmol/kg, with increased thickening occurring with doses up to 224 nmole atRA/kg. Although similar increases in epidermal thickening could be achieved with both 2-methylene-19-nor analogs, CAGE-3 was ineffective in reducing utricle area.

**Fig 4:**
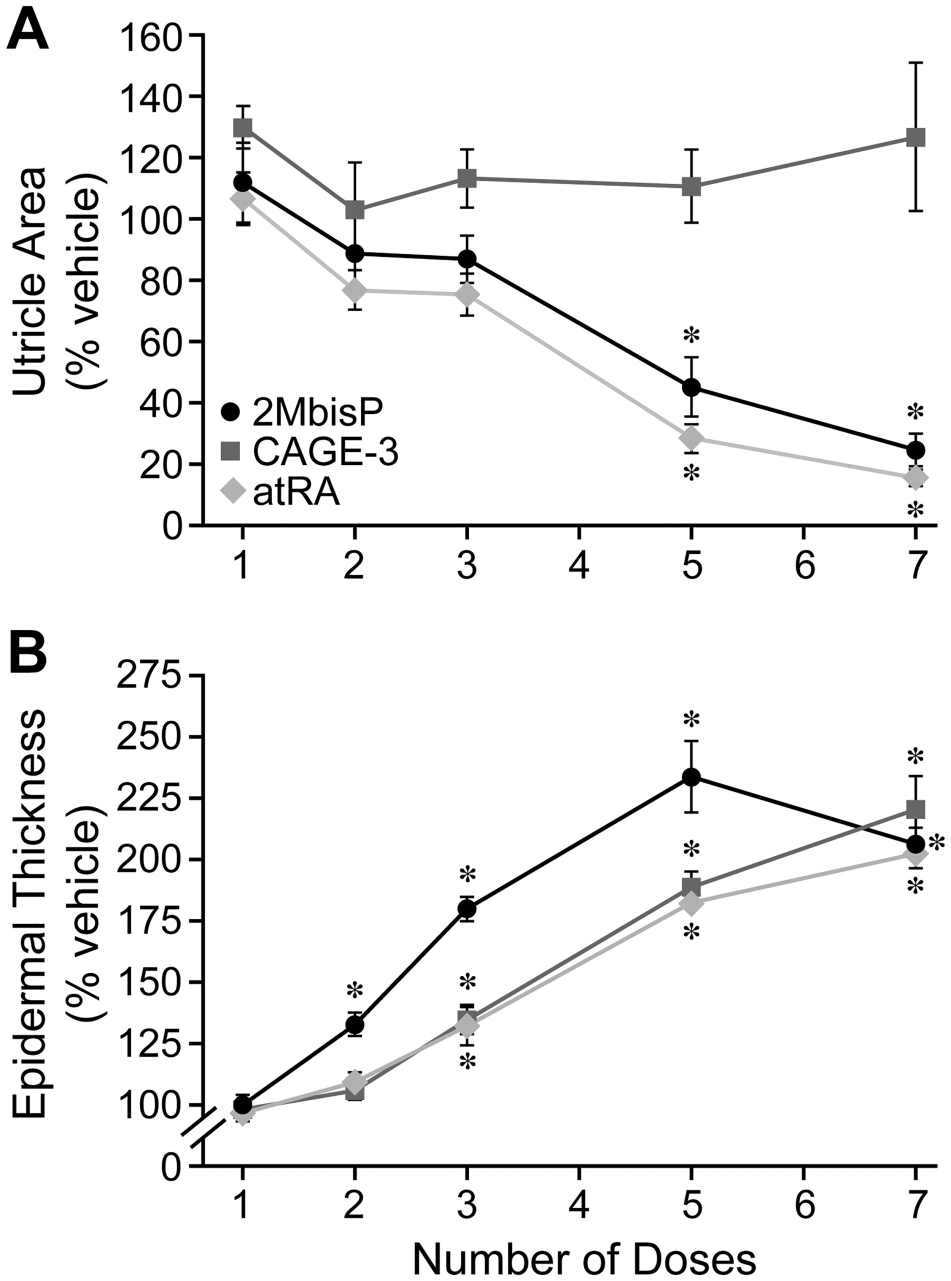
Effect of 1, 2, 3, 5, or 7 doses of 2MbisP, CAGE-3 or atRA on utricle area and epidermal thickness. (A) Utricle area and (B) epidermal thickness were measured in stained tissue sections after the application of vehicle, 2MbisP (690 nmol/kg), CAGE-3 (0.25 nmol/kg body weight), or atRA (224 nmol/kg), and data are expressed as percent of vehicle. Significant differences from the vehicle group at each dosing time point are indicated by an asterisk, *P≤0.05 (S3 Table).

**Fig 5:**
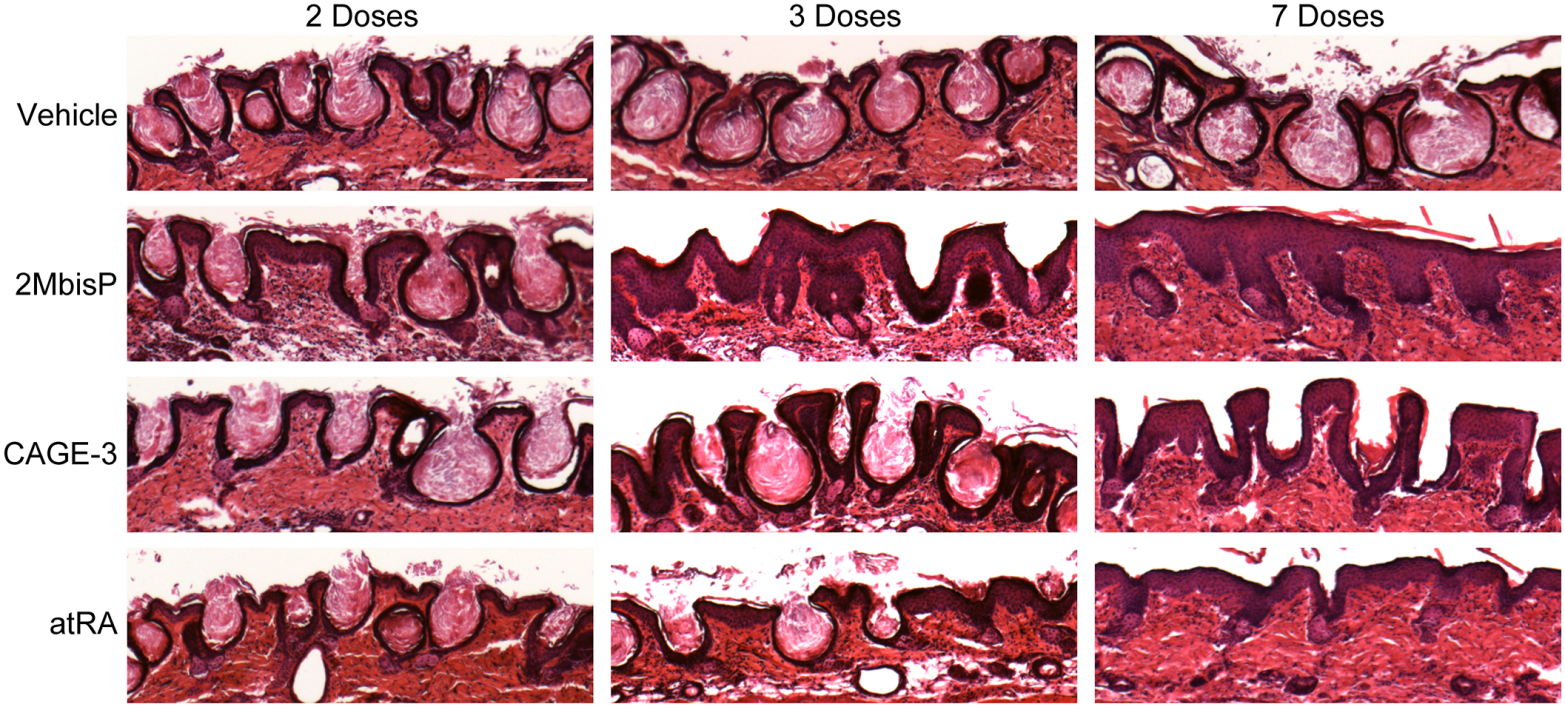
Morphological appearance of the skin after 2, 3 or 7 doses of vehicle, 2MbisP, CAGE-3 or atRA. The drug doses applied are as in Fig 4. Tissue sections were stained with H&E. A representative section for each dosing group is shown. Bar = 0.2 mm.

To determine when skin changes could first be detected, mice were treated topically with 1, 2, 3, 5 or 7 doses of 2-methylene-19-nor analog or atRA, and skin morphology was examined. The dose for 2MbisP and atRA was that maximally effective in reducing utricle size (690 nmol/kg and 224 nmol/kg, respectively), and for CAGE-3 was the highest dose tested and the one that produced the greatest increase in epidermal thickening (0.25 nmol/kg). After 5 doses, both atRA and 2MbisP produced a significant reduction in utricle size (Fig 4A) and after 7 doses, utricle area was further reduced to 25% and 16% of the vehicle group, respectively. Utricle area for CAGE-3 remained unchanged over the course of the experiment. 2MbisP produced a significant increase in thickness of the interfollicular epidermis after only two doses (28 h after the initiation of dosing), whereas both CAGE-3 and atRA required three doses before an increase was observed (Fig 4B). After 7 doses, the increase in epidermal thickness was similar for all three compounds. This work shows that the reduction in utricle area by 2MbisP and atRA occurs over a similar time frame, whereas 2MbisP acts more rapidly than either CAGE-3 or atRA to produce epidermal thickening. A representative image from each treatment group after 2, 3 and 7 doses is shown in Fig 5. The ability of 2MbisP to produce thickening of the epidermis alone cannot account for its ability to reduce utricle size, as CAGE-3 also produced thickening with no reduction in utricle area.

### Effect of 2MbisP and CAGE-3 on EGFR ligand mRNA

**Fig 6:**
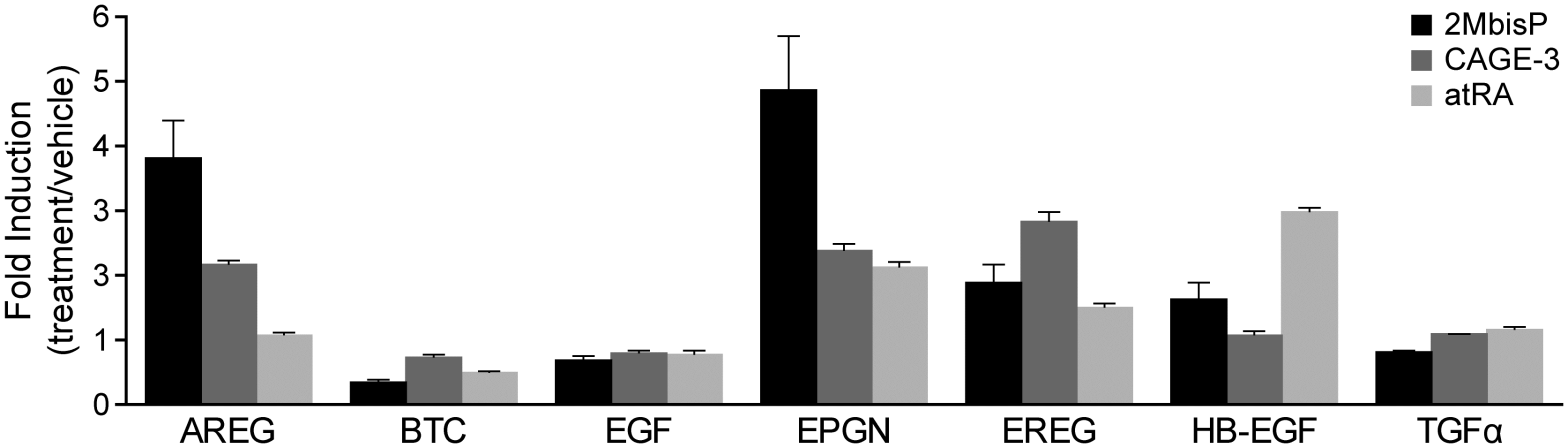
EGFR ligand mRNA after 7 topical doses of 2MbisP, CAGE-3 or atRA. EGFR ligand mRNAs were analyzed by RT-PCR in skin taken 4 h after receiving the final dose of vehicle, 2MbisP (690 nmol/kg), CAGE-3 (0.25 nmol/kg), or atRA (224 nmol/kg) and the data are expressed relative to the respective vehicle-treated group (treatment/vehicle).

To determine whether 2MbisP or CAGE-3 might influence the expression of EGFR ligands, mRNAs for amphiregulin (AREG), betacellulin (BTC), epidermal growth factor (EGF), epithelial mitogen (epigen or EPGN), epiregulin (EREG), HB-EGF, and transforming growth factor alpha (TGFα) were examined in the dorsal skin of mice after 7 days of treatment. The receptor for these ligands, EGFR (ErbB1), was also examined. atRA was included as a control as it has been reported to increase the expression of HB-EGF in mouse skin [36, 49, 50]. The results in Fig 6 show the expected increase in HB-EGF mRNA by atRA, whereas AREG and EPGN but not HB-EGF were most robustly increased by 2MbisP. EREG mRNA was increased by all three of the compounds, whereas EGF, TGF-α and BTC mRNA levels were largely unchanged from vehicle levels. Because an increase in EGFR has been reported in 1,25(OH)_2_D_3_–treated keratinocyte cultures [58], EGFR mRNA was examined but was not changed by 2MbisP, CAGE-3 or atRA when applied for 7 days to rhino skin *in vivo* (S4 Table).

**Fig 7:**
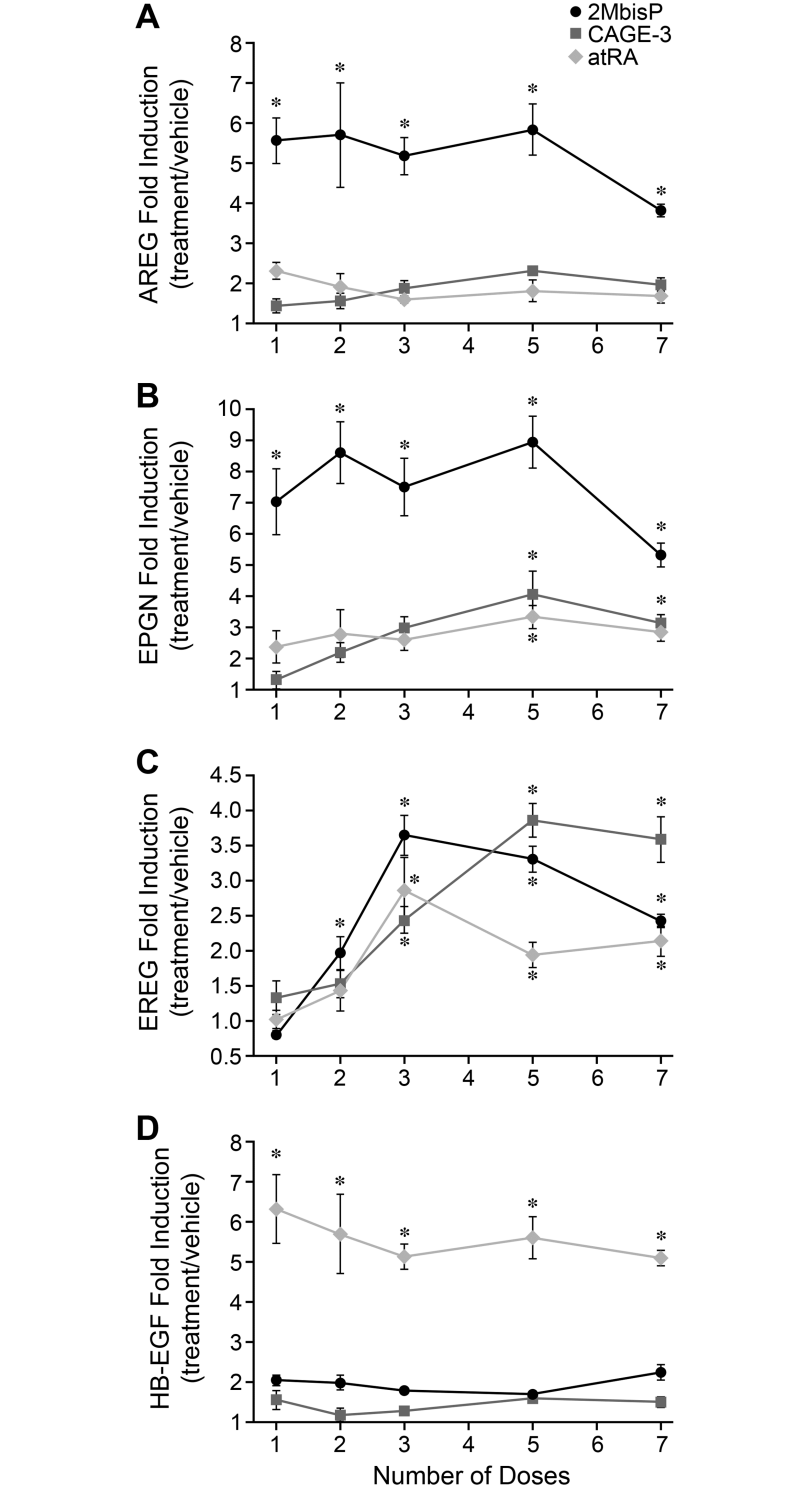
Effect of 1, 2, 3, 5, or 7 doses of 2MbisP, CAGE-3 or atRA on AREG, EPGN, EREG, and HB-EGF mRNA expression in skin. AREG, EPGN, EREG and HB-EGF mRNAs were analyzed by RT-PCR in skin taken 4 h after receiving the final dose of vehicle, 2MbisP (690 nmol/kg), CAGE-3 (0.25 nmol/kg), or atRA (224 nmol/kg). The data are expressed relative to the vehicle-treated group (treatment/vehicle). Significant differences from the vehicle group at each respective dose are indicated by an asterisk, *P≤0.05 (S5 Table).

In order to further probe vitamin D analog effects on AREG, EPGN, EREG, and HB-EGF, transcripts were examined in skin after administering 1, 2, 3, 5 or 7 doses of 2MbisP, CAGE-3, or atRA. As shown in Fig 7A, AREG mRNA was significantly increased by 2MbisP (>5-fold) after only a single dose and remained elevated, whereas this mRNA was not significantly increased above vehicle after treatment with either CAGE-3 or atRA. EPGN mRNA level was increased significantly 4 hours after a single dose of 2MbisP (7-fold), and the increase was maintained at all times studied thereafter (Fig 7B). EPGN mRNA was increased to a lesser extent by CAGE-3 and atRA (2- to 3- fold), and required the administration of 5 doses before a significant increase above vehicle was observed. EREG mRNA level was increased after 2 doses of 2MbisP, and was also increased by CAGE-3 and atRA after 3 or more doses (Fig 7C). HB-EGF mRNA showed the expected induction by atRA (5- to 7- fold), but was unaffected by either 2-methylene-19-nor vitamin D analog. Thus, AREG and EPGN mRNAs appear to be uniquely regulated by 2MbisP, with increases in AREG occurring exclusively in response to 2MbisP, and EPGN induction occurring more rapidly and with a greater magnitude change when compared to CAGE-3 or atRA.

**Fig 8:**
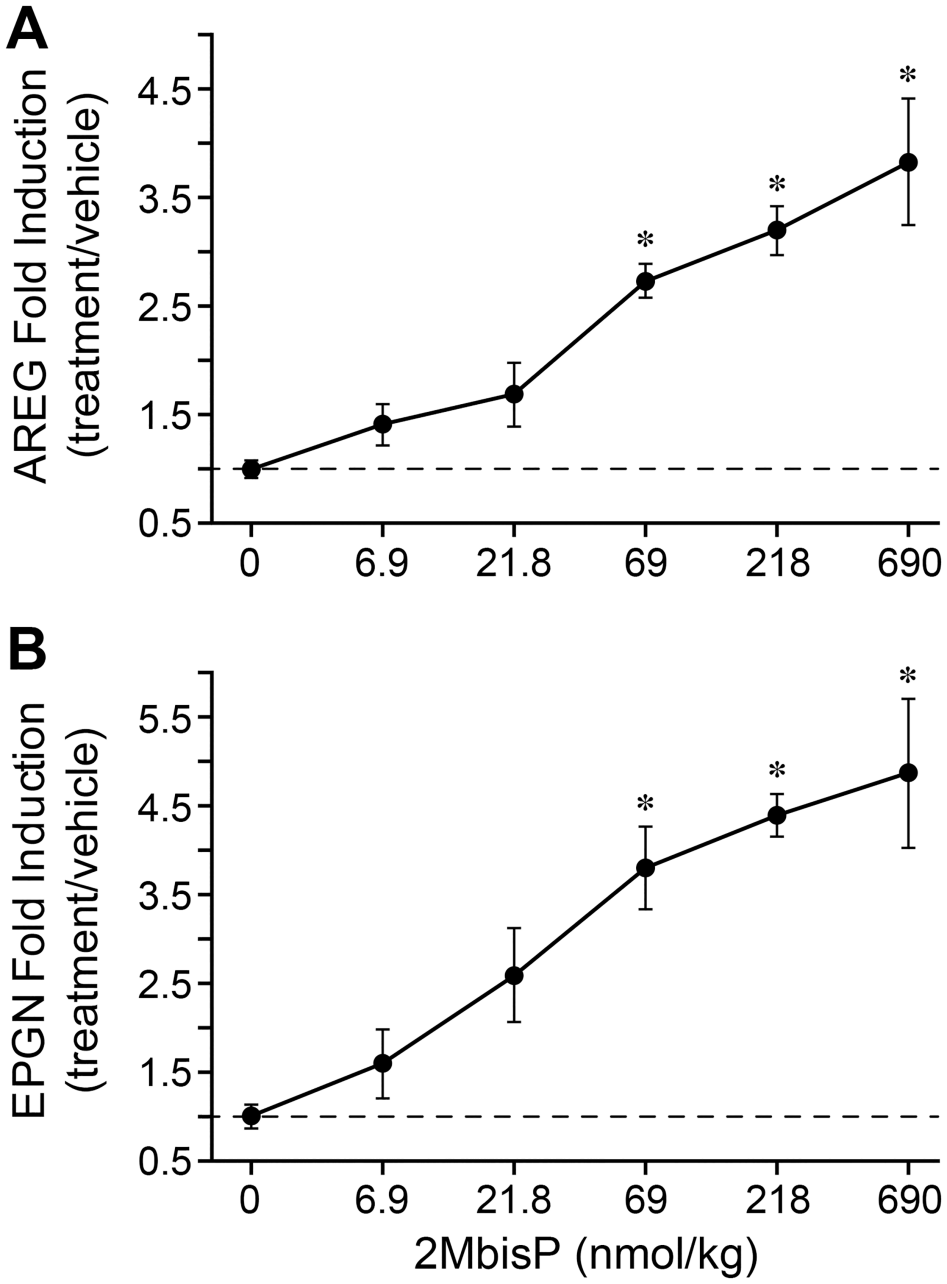
Dose response for 2MbisP induction of AREG and EPGN mRNA. Mice received 7 doses of vehicle or varying doses of 2MbisP and mRNA was quantitated by RT-PCR and expressed as ratio of 2MbisP/vehicle. Significant differences from the vehicle group at the respective dose are indicated by an asterisk, *P≤0.05 (S6 Table).

Because 2MbisP selectively increased AREG and EPGN mRNAs, skin from the dose-response experiment was examined to determine whether doses resulting in changes in transcript corresponded to those also producing biological responses in skin. Both AREG and EPGN mRNAs were induced by 2MbisP, with a significant increase first observed at 69 nmol/kg 2MbisP, and further increases occurring at 218, and 690 nmol/kg (Fig 8). These are the same doses resulting in an increase in epidermal thickness with the two higher doses also producing a significant reduction in utricle area (218 and 690 nmol/kg; compare Fig 3A and 3B with Fig 8). Taken together, this work suggests that the induction of AREG and EPGN mRNAs by 2MbisP could play a role not only in its ability to increase the thickness of the interfollicular epidermis, but that it might also be involved in its activity in reducing utricle size.

### Location of EGFR ligand mRNAs in the skin of mice treated with 2MbisP, CAGE-3, and atRA

**Fig 9:**
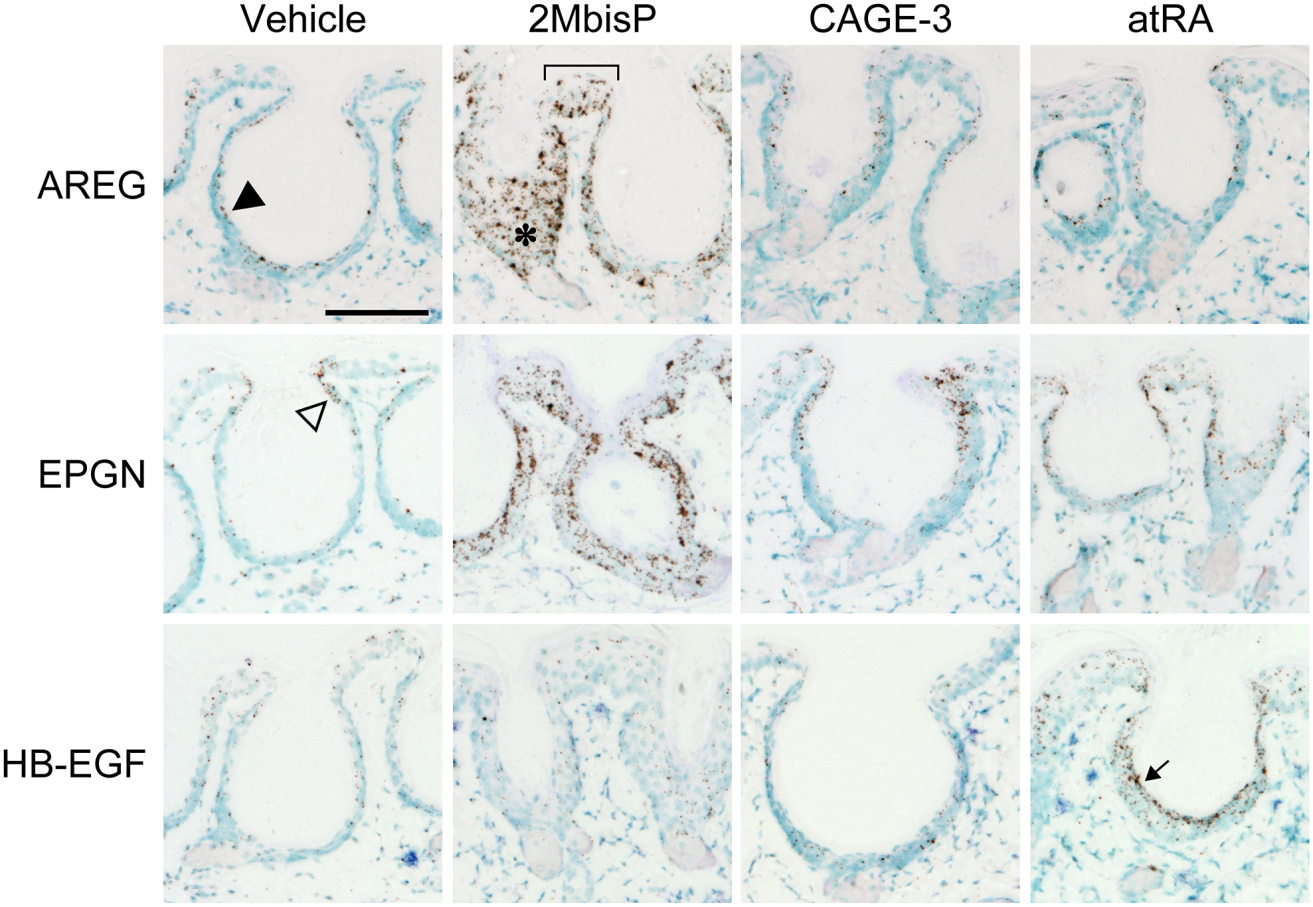
*In situ* hybridization of AREG, EPGN, and HB-EGF mRNA skin treated with vehicle, 2MbisP, CAGE-3 or atRA. Compound or vehicle was applied topically every 24 h, followed by harvesting of the skin 4 h after the third dose. mRNA was detected in paraformaldehyde-fixed tissue sections using RNAscope probes and amplification system, followed by incubation with the substrate, diaminobenzidine, yielding a brown product. Tissue sections were counterstained with methyl green and were analyzed using brightfield microscopy. Bar = 0.1 mm.

In order to determine where AREG and EPGN mRNAs are induced, *in situ* hybridization was performed on skin taken after 3 days of treatment of with vehicle, 2MbisP, CAGE-3 or atRA. HB-EGF was examined as a control. This method enables identification of the region of the stratified epidermis where the EGFR ligand transcripts are being made. Intense AREG mRNA staining was observed in 2MbisP-treated skin (Fig 9). Staining was not confined to the suprabasal layer but extended throughout the entire epidermis in the region of closing utricles (asterisk). Strong AREG staining was also observed in 2MbisP-treated skin in the interfollicular epidermis (bracket) as well as in the basal region at the base of utricles that were fully open. A low level of AREG mRNA staining equivalent to that observed in the vehicle (filled arrowhead) was observed largely in the suprabasal region in CAGE-3 and atRA treated skin. EPGN mRNA was also increased after treatment with 2MbisP, and was observed in both the basal and suprabasal layers in the interfollicular epidermis and closing utricles. Light staining for EPGN mRNA was observed in vehicle, CAGE-3 and atRA treated skin, with most staining observed adjacent to the juncture of the open follicle and the interfollicular epidermis (open arrowhead). atRA-treated skin showed the expected increase in HB-EBF mRNA expression, with the majority of staining confined to the suprabasal region (arrow), whereas, only a low level of HB-EGF staining was seen in the 2MbisP and CAGE-3 groups and was similar to that observed in the vehicle group. Thus, both AREG and EPGN mRNAs were induced by 2MbisP throughout the epidermis.

### VDR expression

**Fig 10:**
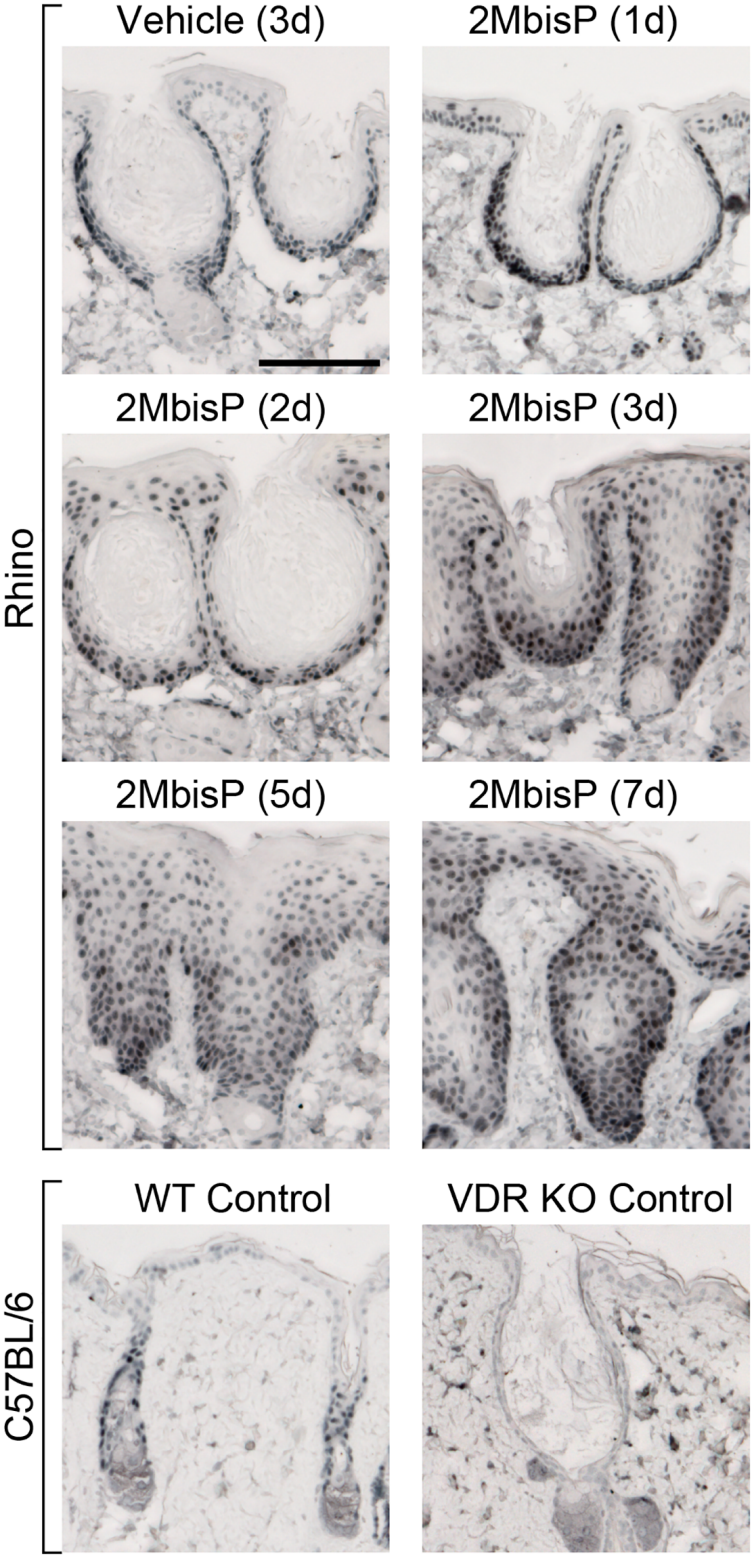
Immunohistochemical analysis of the VDR in skin after treatment with vehicle or 2MbisP. Tissue sections from rhino mice treated with vehicle or 1, 2, 3, 5 or 7 doses (d) of 2MbisP (690 nmol/kg) were probed with antibody to the VDR. Samples from a VDR knock-out mouse and a wild-type litter mate control were studied as controls [59]. Specific staining is observed in the outer root sheath and interfollicular epidermis of the wild-type control mouse. Specific antibody staining is abscent in the epidermis of the VDR knock-out control sample. Light diffuse staining in the dermis of the VDR knock-out, wild type and rhino skin represents non-specific background staining. Bar = 0.1 mm.

The vitamin D receptor (VDR) is a nuclear transcription factor activated by binding to the hormone ligand, 1α,25(OH)_2_D_3_. Both 2MbisP and CAGE-3 bind to the VDR and activate downstream gene expression [53, 54]. In order to determine where the VDR is expressed, immunohistochemistry was performed on skin from vehicle and 2MbisP treated mice. In vehicle treated rhino skin, VDR protein was highest in the basal cells of the epidermis, but was also expressed in the suprabasal keratinocytes (Fig 10). In 2MbisP treated samples, strong staining was found in basal and suprabasal regions of the epidermis separating the utricles (interfollicular region) as well as throughout the closing utricles. VDR was not expressed in the sebaceous cells found at the base of the utricles, and only nonspecific staining was observed in the dermis. Thus, keratinocytes in the basal layer as well as those in the suprabasal region are equipped with the machinery needed to respond to these 2-methylene-19-nor vitamin D analogs.

## Discussion

2MbisP and atRA both increase epidermal proliferation and reduce utricle size in the rhino mouse yet produce very different effects on EGFR ligand mRNA expression. CAGE-3, another 2-methylene-19-nor vitamin D analog, produces epidermal thickening but does not reduce utricle area and does not produce the same array of transcriptional changes in EGFR ligands as 2MbisP. 2MbisP but not CAGE-3 rapidly induces transcripts encoding for two EGFR ligands, AREG and EPGN. The unique set of transcriptional changes induced by 2MbisP may contribute to its ability to both reduce utricle size and cause thickening of the epidermis.

Members of the EGFR ligand family have been associated with an increase in keratinocyte proliferation by activation of EGFR signaling [29, 60]. In the mouse, retinoid-induced hyperproliferation is reported to be largely mediated through induction of HB-EGF in the suprabasal region followed by signaling to the basal cells [36, 49, 50]. Although 2MbisP and atRA both produce epidermal thickening, 2MbisP does not alter the expression of HB-EGF mRNA. Thus, 2MbisP and atRA affect different members of the EGFR ligand family when applied topically to the skin.

Amphiregulin (AREG) is a proteoglycan-dependent ligand of EGFR that influences keratinocyte proliferation [34, 35] and transgenic overexpression in mouse skin leads to a hyperproliferative state [61]. The present work shows that AREG mRNA is rapidly increased throughout the basal and suprabasal layers of the epidermis after exposure to 2MbisP, but that its expression is unchanged after exposure to CAGE-3 or atRA. The lack of atRA effect on AREG mRNA agrees with published work in normal mice [49]. That 2MbisP increases AREG mRNA in both the basal and suprabasal cell layers, combined with the presence of VDR in these regions, suggests this analog could act directly on both proliferating and differentiating keratinocytes.

Both EPGN and EREG have been shown to stimulate human keratinocyte proliferation [62, 63]. The increase in EREG mRNA occurred gradually over the course of dosing with all three compounds and may represent a secondary effect, as this mRNA was found to be induced by various ligands of EGFR including AREG and HB-EGF in cultured keratinocytes [63]. The increase in EPGN mRNA was, however, greater in magnitude and occurred more rapidly in response to exposure to 2MbisP. It is possible that changes in both the EPGN and EREG mRNAs could contribute to increases in epidermal thickness seen with all three compounds.

The vitamin D analog CAGE-3 produces epidermal thickening yet has no effect on AREG mRNA and does not reduce utricle size as seen with 2MbisP. Thus, the absence of a full side chain and the 25-hydroxyl group, although not essential for ligand binding, could be a determinant in tissue-specific response for 2MbisP [64]. The VDR ligand-binding domain also exhibits differential chemical shifts by NMR when bound to structurally distinct VDR analogs [65]. Differing ligand-induced VDR conformations could lead to the activation of distinct transcriptional networks. In addition, cell type-specific factors may also modulate response to distinct receptor-ligand conformations. Although a VDR mechanism seems most likely, it is possible that 2MbisP could exert actions on other pathways. For example, endogenous non-classical vitamin D hydroxylated metabolites, many of which are found in skin, have been reported to influence both the VDR and ROR [66–70].

Stem cells are known to reside both in the basal region of the epidermis as well as the bulge residing at the base of the permanent epithelial portion of the hair follicle [5, 6, 71]. One possibility is that both 2MbisP and CAGE-3 stimulate the proliferation of basal stem cells in the interfollicular region, but 2MbisP may more potently affect a stem cell population that remains in the utricle remnant leading to a filling in of the utricle with new cells.

In closing, we have shown that the short side chain vitamin D analog, 2MbisP, and the retinoid, atRA, both cause thickening of the epidermis and a reduction in utricle size. 2MbisP produces an increase in AREG mRNA throughout the epidermis, whereas atRA causes an increase in HB-EGF in the suprabasal region, revealing these compounds act on skin by different mechanisms. Despite the fact that both 2MbisP and CAGE-3 are 2-methylene-19-nor vitamin D analogs, they exhibit different activity in skin as CAGE-3, unlike 2MbisP, does not reduce utricle size or induce AREG mRNA expression. Thus, 2MbisP is an active vitamin D analog producing a unique profile of EGFR ligand mRNA expression and biological activity in skin.

## Supporting information

S1 Table Statistical summary of two-way ANOVA column and row effects of 2MbisP and atRA on utricle area and epidermal thickness. (XLSX)

S2 Table Statistical summary of one-way ANOVA for dose response of 2MbisP, CAGE-3 and atRA on utricle area and epidermal thickness. (XLSX)

S3 Table Statistical summary of two-way ANOVA column and row effects of 1, 2, 3, 5, or 7 doses of 2MbisP, CAGE-3 or atRA on utricle area and epidermal thickness. (XLSX)

S4 Table EGFR mRNA (fold induction from vehicle) after 7 topical doses of 2MbisP, CAGE-3, or atRA. (XLSX)

S5 Table Statistical summary of two-way ANOVA column and row effects of 1, 2, 3, 5, or 7 doses of 2MbisP, CAGE-3 or atRA on AREG, EPGN, EREG, and HB-EGR mRNA in skin. (XLSX)

S6 Table Statistical summary of one-way ANOVA of dose response for 2MbisP induction of AREG and EPGN mRNA. (XLSX)
